# Gut microbiota dysbiosis in polycystic ovary syndrome: focus on diet, probiotics, and traditional Chinese medicine

**DOI:** 10.3389/fmicb.2025.1659783

**Published:** 2025-11-25

**Authors:** Shuangquan Zhu, Hao Chen, Bing He, Yi Zhang, Ping Li, Jilin Kuang

**Affiliations:** 1 Gynecology Department, The Second Affiliated Hospital of Hunan University of Chinese Medicine, Changsha, Hunan, China; 2 College of Integrative Medicine, Hunan University of Traditional Chinese Medicine, Changsha, Hunan, China; 3 The First Hospital of Hunan University of Chinese Medicine, Changsha, Hunan, China

**Keywords:** polycystic ovary syndrome, gut microbiota, high-fat diets, ketogenic diets, traditional Chinese medicine

## Abstract

Polycystic ovary syndrome (PCOS) is a complex endocrine and metabolic disorder, primarily characterized by symptoms such as ovulatory dysfunction, hyperandrogenism, and polycystic ovarian morphology. In recent years, research has revealed that gut microbiota dysbiosis plays a crucial role in the pathogenesis of PCOS. Diet, as an essential factor in regulating gut microbiota, significantly impacts the clinical presentation and metabolic status of PCOS patients. Although substantial research has explored the relationship between PCOS and gut microbiota, many controversies and gaps remain, including the unclear mechanisms by which dietary structure and nutritional interventions specifically influence PCOS. This review aims to summarize the interaction between PCOS and gut microbiota, explore the role of diet in modulating gut microbiota and improving the pathological state of PCOS, and evaluate the potential therapeutic effects of probiotics, high-fat diets, and ketogenic diets on PCOS. Ultimately, it looks forward to personalized nutritional treatment strategies based on gut microbiota and future research directions, providing new insights into the treatment of PCOS.

## 1 Introduction

Polycystic ovary syndrome (PCOS) is a common endocrine disorder among reproductive-age women that significantly impacts fertility (Deans, 2019). Its primary characteristics include irregular menstrual cycles, increased body hair, ovulatory dysfunction (OD), hyperandrogenism (HA), and polycystic ovary morphology (PCOM) (Dewailly et al., 2016; Li et al., 2019). Currently, there are three definitions of PCOS, with the Rotterdam criteria being the most widely accepted and recognized standard. According to the Rotterdam criteria (Rotterdam ESHRE/ASRM-Sponsored PCOS Consensus Workshop Group, 2004), at least two of the following criteria must be met to diagnose PCOS: Clinical and/or biochemical hyperandrogenism (HA); Ovulatory dysfunction (OD); Polycystic ovarian morphology (PCOM). From 1990 to 2019, the incidence of PCOS increased from 1.4 million to 2.1 million, with the highest incidence globally observed in the 10–19 age group (Zhang et al., 2024). In recent years, with the deepening of research on PCOS, it has been closely related to metabolic disorders, obesity, insulin resistance, and other conditions, and these factors are commonly present in PCOS patients (Chen et al., 2022a; Luo et al., 2022, p. 2). PCOS increases the risk of further complications such as cardiovascular disease (Gao et al., 2023a), type 2 diabetes (Chen et al., 2022b), metabolic syndrome, depression, and anxiety (Damone et al., 2019). Therefore, a thorough understanding of the epidemiological characteristics and clinical manifestations of PCOS is crucial for developing effective prevention and treatment strategies.

**Figure 1:**
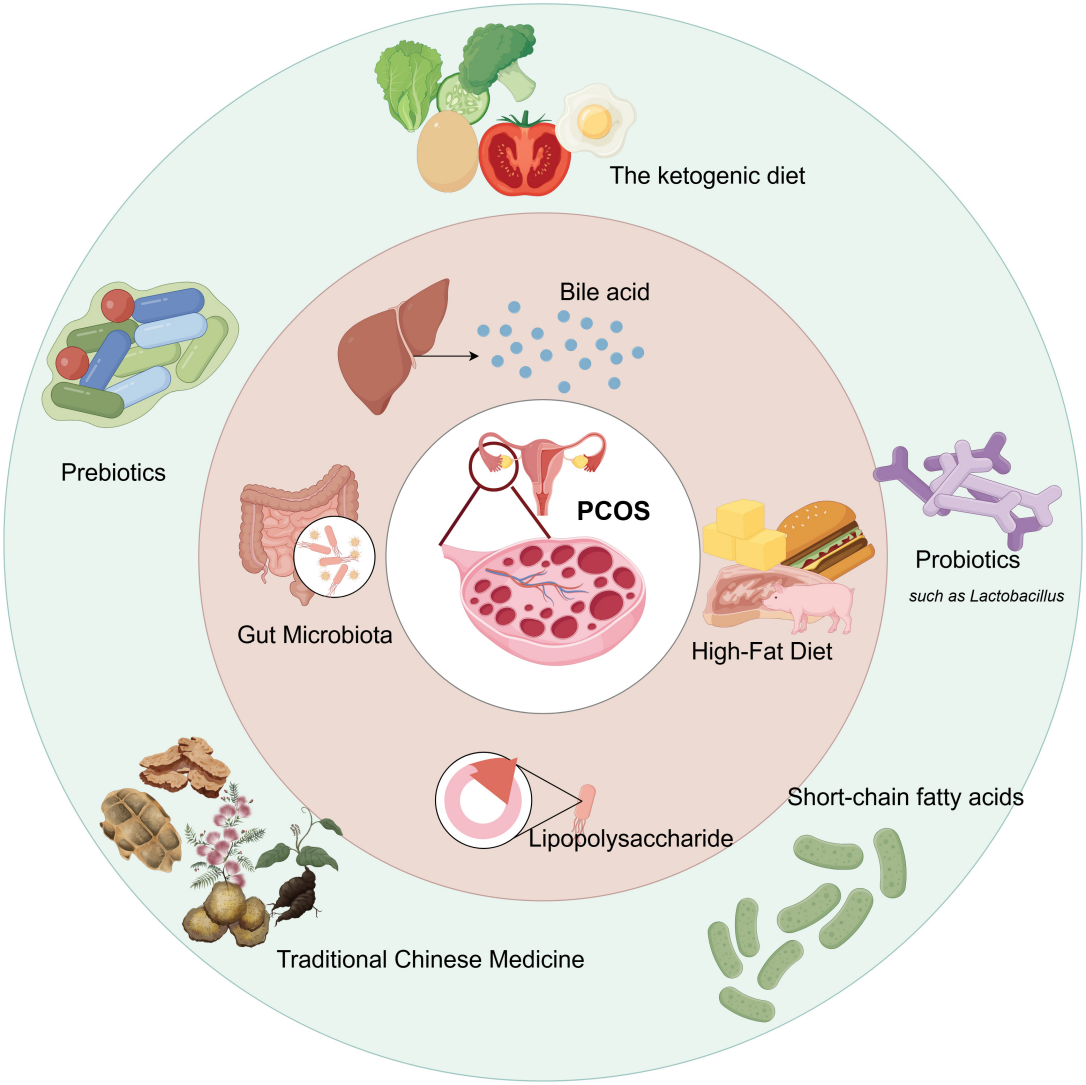
Graphical abstract: PCOS is influenced by dietary structure, gut microbiota, and their metabolic products. Using probiotics, prebiotics, and traditional Chinese medicine to regulate the microbiota, as well as improving the dietary structure, can effectively alleviate PCOS.

The gut microbiota is often referred to as the “second organ” and plays a crucial role in maintaining metabolic and endocrine balance in the human body. The diversity and function of gut microbiota regulate steroid hormone levels through the enterohepatic circulation, including estrogen (Flores et al., 2012; Li et al., 2025c). Studies have found that the gut microbiota composition of PCOS patients differs significantly from that of healthy women, and gut microbiota dysbiosis may further exacerbate metabolic disorders and related complications in PCOS (Lindheim et al., 2017; Torres et al., 2018). Regulating the gut microbiota may represent a novel therapeutic strategy for managing PCOS. Diet, as a key modulator of the gut microbiota, has potential implications for PCOS that should not be overlooked. Studies have shown that a ketogenic diet improves the clinical phenotype and insulin resistance in PCOS rats, while also altering the composition of gut microbiota and metabolites associated with androgen metabolism (Wang et al., 2023). Conversely, a high-sugar, high-fat diet disrupts gut microbiota balance, increases the Firmicutes/Bacteroidetes ratio, and expands the Firmicutes phylum, thereby exacerbating PCOS symptoms (Zheng et al., 2021b). Therefore, optimizing dietary structure and enhancing gut microbiota diversity can help improve metabolic status and physiological function in patients with PCOS. The graphical abstract is shown in Figure 1.

## 2 The role of gut microbiota and their metabolic products in PCOS

### 2.1 Gut microbiota and sex hormones

The gut microbiota is a complex ecosystem composed of approximately 10^1^4 biological species, performing functions such as metabolism and nutrition, antimicrobial protection, maintenance of intestinal mucosal integrity, and regulation of immune responses (Becattini et al., 2016; Sebastián Domingo and Sánchez Sánchez, 2018). Since estrogen levels influence the composition of the microbiota, the gut microbiota composition in women differs significantly from that in men. Studies have shown that the gut microbiota exhibits gender-dependent differences (Wu et al., 2022b; Brown et al., 2023). The gut microbiota plays a crucial role in the onset and progression of PCOS. Clinical studies have shown that compared to healthy women, PCOS patients exhibit lower fecal microbiota diversity, altered gut barrier function, and changes in specific markers of endotoxemia. Hyperandrogenism, total testosterone levels, and hirsutism are negatively correlated with α-diversity (Lindheim et al., 2017; Torres et al., 2018). Compared with non-obese PCOS patients and healthy controls, obese PCOS patients exhibit increased Enterobacteriaceae levels, reduced Lactobacillus and Bifidobacterium levels, and changes in the gut microbiota associated with inflammation and insulin resistance (Zhou et al., 2020). Animal studies have shown that in PCOS mice induced by dehydroepiandrosterone and a high-fat diet, there is a reduction in *Bacteroides* and *Blautia*, and a decrease in *Ruminococcus, Clostridium*, and *Alistipes* (Lin et al., 2021). PCOS mice with impaired glucose and lipid metabolism, hyperinsulinemia, and insulin resistance (IR) exhibited worsened hyperandrogenemia and lipid metabolism disorders following antibiotic mixture intervention (Wang et al., 2022). After fecal microbiota transplantation from PCOS individuals, mice showed insulin resistance, disrupted estrous cycles, increased numbers of cystic follicles, fewer corpus lutea, and elevated levels of testosterone and luteinizing hormone (Qi et al., 2019). In summary, the close relationship between gut microbiota, fecal metabolites, and serum sex hormones influences the progression of PCOS.

The complex interactions between gut microbiota and sex hormones play a significant role in gender differences and PCOS. PCOS leads to excessive production of androgens by the ovaries, resulting in hyperandrogenism, which typically manifests as menstrual irregularities, hirsutism, and acne (Rosenfield, 2024). Hyperandrogenism is associated with the gut microbiota. Studies have shown that PCOS patients commonly exhibit hyperandrogenism, and elevated testosterone levels can alter gut microbiota structure by regulating bile acid signaling pathways (such as FXR, CYP7A1, etc.), leading to a reduction in the abundance of metabolically protective microbiota such as *Akkermansia* (Duan et al., 2024). Dehydroepiandrosterone (DHEA) induces gut microbiota dysbiosis in PCOS rats. Eliminating the gut microbiota with an antibiotic cocktail does not prevent the development of PCOS phenotypes. Transplanting PCOS rat gut microbiota into germ-free rats disrupts hepatic glucose and lipid metabolism and causes reproductive hormone imbalances (Han et al., 2021). Hyperandrogenemia may lead to insulin resistance and PCOS metabolic abnormalities by causing intestinal bacterial enrichment. This microbiota dysbiosis exacerbates insulin resistance, forming a vicious cycle of “hyperandrogenism-microbiota dysbiosis-metabolic abnormalities” (Zeng et al., 2019). Androgens are produced by theca cells, while estrogens are produced by granulosa cells and can be interconverted within the body. Gut microbiota can interfere with the enterohepatic circulation of sex hormones by secreting enzymes, such as β-glucuronidase, which further increases serum free testosterone levels (Cross et al., 2024). Gut microbial β-glucuronidase is a key factor in regulating host estrogen metabolism, as it activates estrogen. Additionally, estrogen levels also influence the composition and diversity of the gut microbiota (Ervin et al., 2019). Studies have found that abnormalities in the estrogen receptor signaling pathway may weaken the microbiota's protective role in the intestinal barrier, allowing endotoxin to enter the bloodstream and induce chronic, low-grade inflammation (Kumari et al., 2024). In summary, gut microbiota dysbiosis not only affects the metabolism and levels of sex hormones but also exacerbates PCOS symptoms. Improving gut microbiota health may emerge as a new therapeutic strategy for PCOS, helping to regulate hormone levels, alleviate symptoms, and enhance patients' quality of life.

Insulin resistance (IR) and the resulting compensatory hyperinsulinemia are core metabolic abnormalities in polycystic ovary syndrome (PCOS), particularly in obese phenotypes. Alterations in the microbial metabolite profile disrupt bile acid pools and short-chain fatty acid levels, impair mucosal energy supply and intestinal barrier integrity, and modulate incretin hormone secretion, thereby altering FXR and GPBAR1 signaling in the intestine and liver, ultimately leading to IR (He and Li, 2020; Yang et al., 2021). In parallel, Gram-negative bacterial overgrowth elevates lipopolysaccharide (LPS) translocation, triggering low-grade endotoxemia and macrophage infiltration in adipose and ovarian tissue; this inflammatory milieu further amplifies insulin resistance and hyperandrogenism (Lindheim et al., 2017). Clinical microbiome studies confirm that the microbial composition of insulin-resistant PCOS patients differs significantly from that of non-IR individuals, underscoring a distinct microbial signature associated with metabolic impairment (Torres et al., 2018). Thus, IR occupies a central position at the intersection of metabolic, endocrine, and microbial axes in PCOS. Beyond androgens and insulin, other sex steroids—estrogens and progesterone—also exert bidirectional interactions with the gut microbiota. Dysbiosis, characterized by low microbial diversity or reduced estrobolome activity, decreases circulating free estrogens and alters the estrogen-to-androgen ratio, thereby affecting reproductive and metabolic homeostasis (Li et al., 2025c). Conversely, fluctuating estrogen and progesterone levels throughout the menstrual cycle, pregnancy, or menopause reshape the intestinal microbiota, influencing mucosal permeability, motility, and immune tone (Atoum and Padma, 2025; Li et al., 2025a). Restoration of a healthy gut ecosystem—through dietary modulation, probiotics, or targeted microbial therapies—may therefore offer a promising strategy to alleviate insulin resistance, rebalance sex hormones, and improve clinical outcomes in PCOS.

### 2.2 Gut microbiota metabolites and PCOS

Gut microbiota metabolites such as bile acids (BA), short-chain fatty acids (SCFA), branched-chain amino acids (BCAA), and trimethylamine N-oxide (TMAO) can be produced from food and interact with gut bacteria in the body, and are associated with the onset and progression of PCOS (Chen and Pang, 2021). Clinical studies have found that lean women with PCOS have significantly increased levels of chenodeoxycholic acid (CDCA), which is positively correlated with total testosterone and free androgen index (Zhu et al., 2024). In PCOS patients, serum CDCA and LCA levels are significantly elevated, and DCA is associated with the sedimentation index, fasting, and postprandial insulin levels, and is influenced by changes in testosterone (Yu et al., 2023). PCOS patients exhibit significantly elevated levels of glycine- and taurine-conjugated primary bile acids, and the increase in circulating conjugated primary bile acids is positively correlated with hyperandrogenism in PCOS women (Zhang et al., 2019a). Alterations in the bile acid profile are a risk factor for hyperandrogenism in PCOS patients. Animal studies have confirmed that women with PCOS have reduced levels of glycocholic acid and taurocholic acid. Transplantation of fecal microbiota from PCOS women or *Bacteroides vulgatus* into mice resulted in increased ovarian dysfunction, insulin resistance, and altered bile acid metabolism (Qi et al., 2019). *Bacteroides vulgatus* also acts independently of bile acids via its metabolite guanidine, activating the farnesoid X receptor (FXR) pathway, which subsequently inhibits L-cell secretion of glucagon-like peptide-1 (GLP-1), leading to insulin resistance and ovarian dysfunction and thereby causing PCOS-like symptoms (Yun et al., 2024). In summary, elevated levels of *Bacteroides vulgatus* in the gut microbiota are associated with altered bile acid metabolism in PCOS.

SCFAs influence PCOS by regulating glucose metabolism or hormonal signaling, and specific gut microbiota, fecal fatty acids, and serum metabolites may mediate the onset and progression of PCOS. The Clostridiaceae, Erysipelotrichidae, Lachnospiraceae, Lactobacillaceae, and Ruminococcaceae can produce SCFAs [37]. Studies have found that PCOS patients exhibit reduced levels of *Faecalibacterium, Bifidobacterium*, and *Blautia* in their gut microbiota, suggesting an association between PCOS and the reduction of SCFA-producing bacteria (Zhang et al., 2019b). PCOS rats exhibit reduced β-diversity and decreased butyrate-producing bacteria. Regulating the butyrate-dependent gut-brain-ovary axis to improve PCOS is effective (Feng et al., 2022). Following sleeve gastrectomy, PCOS rats exhibited increased abundance of Bacteroides and Blautia, along with reduced levels of fecal SCFAs, particularly butyrate, which significantly improved PCOS-related symptoms such as hyperandrogenism, ovarian dysfunction, and impaired glucose tolerance (Lin et al., 2021). It was found that serum butyrate levels in obese PCOS patients were lower compared to other groups. Butyrate can alleviate inflammation in granulosa cells by regulating mRNA modifications through the METTL3-mediated N6-methyladenosine (m6A) pathway. Supplementing with butyrate improves ovarian function and reduces the expression of local inflammatory factors in the ovaries (Liu et al., 2023). The levels of acetate and propionate in the feces of PCOS patients were significantly higher than those in healthy controls (Li et al., 2022). Compared with other SCFAs such as acetate and propionate, butyrate plays a significant role in alleviating PCOS symptoms (He et al., 2022b). In summary, SCFAs, particularly butyrate, play a crucial role in regulating the gut microbiota composition, reducing inflammation, and alleviating PCOS symptoms.

### 2.3 Endotoxins and inflammatory responses

The metabolic pathways of the gut microbiota play a significant role in regulating systemic inflammation, particularly through short-chain fatty acids (SCFAs), which help maintain glucose and insulin homeostasis and alleviate systemic chronic inflammation. SCFAs can significantly improve insulin sensitivity, inhibit chronic inflammatory responses, and thereby promote overall health by regulating the composition and activity of the gut microbiota (Chen et al., 2023). Additionally, IL-22, a key cytokine, plays a crucial role in regulating insulin sensitivity and lipid metabolism. Reduced IL-22 levels disrupt the integrity of the intestinal barrier and the microbiota's hemostatic function, thereby exacerbating endotoxemia and chronic inflammatory states (Kriebs, 2019; Qi et al., 2020). Patients with PCOS typically exhibit chronic, low-grade inflammation, characterized by significantly elevated circulating C-reactive protein levels, indicating persistent inflammatory responses (Aboeldalyl et al., 2021). Reduced IL-22 levels in PCOS individuals, while IL-22 administration improves insulin resistance, menstrual cycle disorders, and ovarian morphological abnormalities, making it a potential treatment for PCOS with hyperandrogenism phenotypes (Qi et al., 2020). The possible mechanism by which IL-22 regulates insulin resistance and ovarian dysfunction associated with PCOS may be related to the suppression of inflammation following BA administration (Qi et al., 2019).

Regarding the potential mechanisms underlying PCOS, studies have shown that BA administration can improve insulin resistance and ovarian dysfunction by inhibiting inflammatory responses. This mechanism of action may be closely related to the repair of the intestinal barrier and the regulation of the microbiota. When the intestinal mucosal barrier is damaged, endotoxins, such as lipopolysaccharides, can enter the bloodstream, leading to chronic inflammation (Rooks and Garrett, 2016). Butyrate can increase the expression of tight junction proteins in intestinal epithelial cells and reduce epithelial cell death, thereby promoting intestinal mucosal immunity and barrier integrity (Mathewson et al., 2016). SCFAs may play a role in repairing intestinal mucosal damage and improving PCOS inflammation. Studies have found that PCOS rats have reduced SCFA absorption, increased fecal SCFA concentrations, and positive correlations with tumor necrosis factor and IL-6 levels. Enhancing SCFA absorption can improve intestinal mucosal barrier integrity and inhibit intestinal and extraintestinal inflammation (Lin et al., 2021). Therefore, the critical role of SCFAs and BAs in regulating PCOS-related inflammation and insulin resistance suggests that improving intestinal microbiota metabolic pathways and repairing intestinal barrier function may provide new directions for PCOS treatment.

## 3 The regulatory effects of diet on gut microbiota and PCOS

### 3.1 High-fat diet in PCOS

Dietary fat has been shown to alter gut microbiota composition, with high-fat diets reducing the abundance of the Bacteroidetes phylum while increasing that of the Firmicutes and Proteobacteria phyla (Hildebrandt et al., 2009; Zhang et al., 2012). Changes in the ratio of Firmicutes to Bacteroidetes may be associated with obesity and weight loss following dietary intervention (Ley et al., 2006). Obesity-related gut microbiota dysbiosis is directly linked to a high-fat diet (HFD), which promotes a microbiota similar to that found in obese male mice (de La Serre et al., 2010). The disrupted gut microbiota caused by the HFD is also considered a major mediator of the link between obesity-related diseases and the primary pathological conditions associated with their development (inflammation, hyperandrogenism, and lipid metabolism disorders) (Cani et al., 2009; Zheng et al., 2021a; Wang et al., 2022). Gut microbiota diversity is a crucial factor in maintaining health, and its reduction is often associated with the onset of various metabolic diseases. Studies have shown that HFD leads to significant changes in gut microbiota diversity and composition, characterized by a substantial reduction in diversity, a decrease in beneficial bacteria such as *Lactobacillus*, and an increase in inflammation-associated microbiota, including certain Firmicutes and Bacteroidetes (Tan et al., 2025). The decline in *Lactobacillus* may be associated with metabolic disorders and inflammatory responses induced by a high-fat, high-sugar diet. *Lactobacillus* is generally considered a beneficial gut microorganism that maintains a healthy gut environment by producing lactic acid and other short-chain fatty acids (SCFAs) (He et al., 2020). When its abundance decreases, it may lead to an imbalance in the gut environment, increasing the risk of conditions such as metabolic syndrome and insulin resistance (Liow et al., 2024).

HFD not only alters the composition and abundance of the gut microbiota but also influences its metabolic functions by modifying the gut environment. HFD can also lead to dysbiosis of bacterial communities, which, in turn, weakens intestinal barrier function, allowing endogenous pathogenic bacteria and their metabolic products (such as endotoxins) to enter the bloodstream and trigger systemic inflammatory responses more readily (Crawford et al., 2019). Chronic low-grade inflammation is considered a key factor in the development of PCOS and is closely associated with insulin resistance (Barber et al., 2016; Cai et al., 2025). These bacteria may promote inflammation and metabolic disorders by producing active metabolites, further exacerbating the pathological state of PCOS (Gautam et al., 2024; Guan et al., 2024). Dysbiosis of the gut microbiota not only alters its composition but also disrupts its function. Studies have shown that a high-fat diet significantly reduces the abundance of SCFA-producing bacterial genera in the gut, including *Roseburia* and *Faecalibacterium*, which are the primary producers of butyrate and propionate (David et al., 2014). The reduction in SCFAs is closely associated with symptoms such as insulin resistance, obesity, and endocrine disorders in PCOS patients (Geng et al., 2025). Recent studies have shown that SCFAs, such as acetate and butyrate, can improve ovarian lipid metabolism and endocrine dysfunction in PCOS models (Olaniyi and Areloegbe, 2024; Olaniyi et al., 2025). Overall, the changes in gut microbiota diversity and composition induced by a high-fat diet are not only an important component of PCOS pathogenesis but also a potential target for future treatment. Through dietary intervention, probiotics, and other microbiota-modulating strategies, it is possible to improve metabolic and endocrine function in patients with PCOS, thereby enhancing their quality of life and fertility (Ju et al., 2025). Therefore, restoring the balance and diversity of the gut microbiota may provide new strategies and directions for managing and treating PCOS.

### 3.2 Ketogenic diet in PCOS

The ketogenic diet (KD) is a very-low-carbohydrate dietary pattern. Unlike an unrestricted high-fat diet, which typically promotes metabolic imbalance (as depicted in Figure 1), the KD couples controlled lipid intake with markedly reduced carbohydrate intake, thereby lowering circulating glucose and insulin levels and improving overall metabolic homeostasis (Balestra et al., 2025). The ketogenic diet enhances gut health by modifying the gut microbiota composition, promoting the growth of beneficial bacteria, and suppressing the activity of harmful bacteria (Palmas et al., 2025). KD is commonly used to treat neurological disorders. Still, research suggests that it may be an effective strategy for treating metabolic disorders, type 2 diabetes mellitus (T2DM), obesity, and non-alcoholic fatty liver disease, primarily by reducing fat accumulation and inhibiting postprandial insulin secretion (Cunha et al., 2020; Watanabe et al., 2020). The Italian Association of Dietetics and Clinical Nutrition, along with the Italian Society of Obesity, proposes the Very-low-calorie ketogenic diet (VLCKD) as a treatment option for NAFLD and obesity-related comorbidities (Caprio et al., 2019). Clinical trials have confirmed that overweight women with polycystic ovary syndrome (PCOS) who followed a high-protein, very-low-calorie diet for 4 weeks experienced significant reductions in fasting blood glucose levels and insulin levels, as well as a significant increase in insulin sensitivity (Andersen et al., 1995). The ketogenic diet can significantly improve weight management and insulin sensitivity, which is particularly important for PCOS patients, as PCOS is often associated with insulin resistance and obesity (Paoli et al., 2020). High-fat KD and VLCKD have shown beneficial effects on body weight, body composition, glucose metabolism parameters, and hormone profiles in women with PCOS. VLCKD has a better advantage in reducing fat mass and lowering triglyceride levels (Cannarella et al., 2025). The ketogenic diet also significantly affects hormone metabolism. A 12-week VLCKD intervention in obese, non-diabetic women with PCOS and regular menstrual cycles resulted in a significant decrease in serum anti-Müllerian hormone levels, a significant increase in progesterone and serum sex hormone-binding globulin levels, and VLCKD may also be beneficial for ovarian reserve and luteal function (Magagnini et al., 2022). Short-term KD treatment effectively regulates androgen levels in overweight/obese PCOS patients, reduces total testosterone levels, improves insulin resistance and glucose/lipid metabolism, and significantly reduces body weight (Li et al., 2025b). Compared with a carbohydrate-based diet model, KD demonstrates superior efficacy in improving BMI, weight, blood glucose, insulin, and free testosterone levels (Sharifi et al., 2024). Therefore, the ketogenic diet may offer a novel therapeutic approach for PCOS patients as an intervention strategy.

### 3.3 Mediterranean, DASH, and high-protein diets in PCOS

Beyond ketogenic or very-low-carbohydrate interventions, several other nutritional patterns, namely the Mediterranean diet (MedDiet), the Dietary Approaches to Stop Hypertension (DASH) diet, and high-protein diets (HPD), have emerged as critical dietary strategies for managing polycystic ovary syndrome (PCOS). These diets share overlapping mechanisms that target insulin resistance, oxidative stress, inflammation, and dysbiosis of the gut microbiota. The Mediterranean diet, characterized by abundant consumption of vegetables, fruits, legumes, whole grains, nuts, fish, and extra-virgin olive oil, with minimal processed or red meat, exerts multiple metabolic benefits through its high fiber and polyphenol content (Estruch et al., 2018; Rosato et al., 2019). Randomized controlled trials have demonstrated that an 8-week DASH intervention significantly improved HOMA-IR, hs-CRP, and abdominal adiposity in overweight and obese women with PCOS (Asemi and Esmaillzadeh, 2015). A 12-week trial further reported significant reductions in BMI, AMH, and oxidative stress markers, along with increased SHBG and decreased free androgen index (Foroozanfard et al., 2017).

The DASH diet, initially designed for blood-pressure control, is an isocaloric, nutrient-dense pattern rich in fruits, vegetables, low-fat dairy, legumes, nuts, and whole grains while limiting sodium, red meat, and added sugars (Sacks et al., 2001; Soltani et al., 2016). Randomized controlled trials have demonstrated that an 8-week DASH intervention significantly improved HOMA-IR, hs-CRP, and abdominal adiposity in overweight and obese women with PCOS (Asemi and Esmaillzadeh, 2015). A 12-week trial further reported significant reductions in BMI, AMH, and oxidative stress markers, along with increased SHBG and decreased free androgen index (Foroozanfard et al., 2017). Additional studies confirmed improvements in insulin metabolism, lipid profile, and body composition (Azadi-Yazdi et al., 2017). A recent network meta-analysis (2024) ranked the DASH diet as the most effective dietary pattern overall for metabolic and hormonal regulation in PCOS (Juhász et al., 2024). HPD providing approximately 25–35% of total energy from protein can improve satiety, preserve lean mass, and attenuate postprandial insulin secretion. In a controlled clinical trial, a hypocaloric high-protein/low-GI diet achieved greater improvements in insulin sensitivity and hs-CRP than a conventional low-protein diet (Homeira et al., 2012). A recent systematic review (2024) suggested that HPDs improve insulin resistance and body composition in PCOS patients, though outcomes depend on protein source, fiber content, and energy balance (Wang et al., 2024, 2025).

In summary, while the Mediterranean and DASH diets primarily exert their benefits through gut microbiota modulation, SCFA enhancement, bile acid signaling, and anti-inflammatory effects, high-protein diets mainly regulate satiety, weight, and insulin dynamics.

## 4 Advances in dietary intervention and gut microbiota regulation

### 4.1 Applications of probiotics and prebiotics

Gut microbiota has been shown to play a crucial role in the pathogenesis of PCOS. Therapies targeting the gut microbiota, such as probiotics, prebiotics, or fecal microbiota transplantation, offer promising treatment strategies for PCOS and its associated metabolic disorders (Duan et al., 2021). In terms of hormone regulation, probiotics influence sex hormone levels by modulating the gut microbiota and its metabolites. *Lactiplantibacillus plantarum* alleviates ovarian pathological changes, restores testosterone and luteinizing hormone levels, and increases the abundance of butyrate- and short-chain fatty acid-associated bacteria, such as *Lachnospira* and *Ruminococcus*, thereby alleviating PCOS (He et al., 2022a). Lactic acid bacteria improve gut dysbiosis in PCOS rat models by modulating the abundance of *Akkermansia, Roseburia, Prevotella, Staphylococcus*, and *Lactobacillus* species, which are associated with sex hormone levels, thereby helping to alleviate PCOS (He et al., 2020). Therefore, probiotics have significant potential to regulate gut microbiota, restore hormonal balance, and relieve symptoms of PCOS.

Probiotic supplements have been shown to improve the metabolic status of patients affected by PCOS. Clinical studies have found that *Bifidobacterium lactis V9* significantly reduces LH and LH/FSH levels in PCOS patients, while also considerably increasing sex hormone and gut SCFA levels and regulating sex hormone levels by modulating the gut microbiome (Zhang et al., 2019b). Probiotic supplementation increased serum sex hormone-binding globulin (SHBG) levels by a considerable amount in PCOS patients, normalized menstrual cycles, and significantly decreased serum total testosterone, demonstrating the efficacy of multi-strain probiotics, along with dietary and lifestyle changes, in treating PCOS (Karamali et al., 2018; Kaur et al., 2022). Several clinical studies have confirmed that probiotics and synbiotic supplements improve insulin resistance and serum insulin levels in patients with PCOS [83], reduce HOMA-IR, fasting blood glucose, and blood lipid levels, and lower total testosterone; synbiotics show more pronounced effects than probiotics or prebiotics alone (Shamasbi et al., 2020; Martinez Guevara et al., 2024). Probiotic supplementation also significantly reduces weight, BMI, insulin levels, HOMA-IR, triglycerides, hirsutism, and total testosterone levels in PCOS patients, without affecting dehydroepiandrosterone sulfate (DHEAS) levels, as well as total cholesterol, LDL cholesterol, and HDL cholesterol (Tabrizi et al., 2022). In summary, probiotic supplementation not only improves metabolic disorders but also effectively regulates hormone levels, making it a promising adjunctive treatment for PCOS.

### 4.2 Traditional Chinese medicine and gut microbiota regulation

Traditional Chinese medicine (TCM) plays an increasingly important role in managing PCOS, not only by modulating endocrine and metabolic abnormalities but also by restoring gut-microbiota balance and reducing chronic inflammation. Recent studies integrating network pharmacology, metabolomics, and gut-microbiota analysis have elucidated the multi-target mechanisms of several classical and emerging TCM prescriptions (Kwon et al., 2020).

Cangfu Daotan Decoction (CFDTD), a representative formula for resolving phlegm and dampness, has been shown to improve ovulation rate, insulin sensitivity, and hormonal balance. A meta-analysis confirmed its efficacy and safety in the treatment of PCOS (Wu et al., 2022a). Guizhi Fuling Wan (GZFLW), traditionally used to promote blood circulation and remove stasis, enhances insulin sensitivity and reduces systemic inflammation by regulating gut-microbiota composition and suppressing inflammatory cytokines (Zhu et al., 2020). Mechanistically, GZFLW inhibits granulosa-cell autophagy and promotes follicular development via activation of the PI3K/AKT/mTOR signaling pathway (Liu et al., 2021). The Yulin Tong Bu formula (YLTB) restores metabolic and endocrine homeostasis by recalibrating gut-microbiota structure and fecal metabolites. In animal models, YLTB improved ovarian morphology, enhanced insulin sensitivity, and corrected glucose–lipid metabolism; metabolomic analysis identified ferulic acid as a key mediator of these effects (Su et al., 2023). The herb pair Banxia-Chenpi in the Banxia Xiexin Decoction formula reduces weight gain, normalizes sex hormone levels, and regulates the estrous cycle by activating the CYP17A1-centered steroid biosynthesis pathway (Shen et al., 2025). Dingkun Pill (DKP) significantly improves reproductive and metabolic function in PCOS, lowering testosterone and the LH/FSH ratio, and normalizing folliculogenesis through CYP17A1-Mediated modulation of androgen synthesis (Cai et al., 2022). A systematic review and meta-analysis further verified its clinical efficacy and safety (Jin et al., 2022). The modified Guishen pill significantly improved insulin resistance, apoptosis, and oxidative stress in PCOS rats by regulating sex hormone levels and alleviating ovarian pathological changes in rats (Tian et al., 2022). Traditional Chinese medicine has demonstrated unique advantages in the treatment of PCOS and holds promise as a potential resource for developing novel therapeutic agents.

Traditional Chinese medicine can also exert therapeutic effects on PCOS by improving the structure of the intestinal microbiota and regulating the intestinal microecological environment. Erchen decoction (ECD) enhances ovarian function by regulating the expression of CYP17A1, HSD11B1, AKR1D1, and HSD11B2 in the steroid hormone biosynthetic pathway, thereby improving hormone levels and follicle development (Zhang et al., 2025). HeQi San (HQS) reverses abnormal hormone elevation, improves insulin resistance, and reduces histopathological changes in ovarian tissue, thereby modulating the abundance of gut microbiota in PCOS mice, and may be a potential candidate for PCOS treatment (Li et al., 2024). Angelica sinensis regulates hormonal and lipid metabolism disorders by modulating PI3K/AKT, PPAR, MAPK, AMPK, and insulin signaling pathways in ovarian tissue, as well as maintaining gut microbiota homeostasis, thereby alleviating PCOS (Gao et al., 2023b). The Bushen Huatan formula may improve PCOS by regulating the gut microbiota to increase short-chain fatty acid levels, thereby activating the intestinal PPARγ pathway and improving intestinal barrier function (Cui et al., 2023). The Yulin Tong Bu formula reduces insulin resistance, improves ovarian dysfunction, lipid and glucose metabolism, and hormonal imbalances, and significantly alleviates PCOS-related gut microbiota dysbiosis (Su et al., 2023). In summary, traditional Chinese medicine demonstrates its potential in PCOS treatment by regulating the gut microbiota and improving endocrine imbalance through multiple pathways (Table 1). Although current research findings provide strong evidence, the specific mechanisms require further experimental validation and clinical studies to ensure safety and efficacy.

**Table 1:** Representative TCM formulas for PCOS: mechanisms and effects.

**Formula**	**Main mechanisms**	**Principal therapeutic effects**	**Key targets**	**References**
Cangfu Daotan Decoction (CFDTD)	Resolves phlegm-dampness, modulates inflammation	↑ Ovulation rate; ↑ insulin sensitivity; hormone balance	/	Wu et al., 2022a,b
Guizhi Fuling Wan (GZFLW)	Regulates microbiota; suppresses inflammation; activates PI3K/AKT/mTOR	↓ IR; ↓ inflammation; ↑ follicular development	PI3K/AKT/mTOR	Zhu et al., 2020; Liu et al., 2021
Yulin Tong Bu formula (YLTB)	Recalibrates gut microbiota and metabolites	↑ Ovarian function; ↑ insulin sensitivity; ↓ dysbiosis	SCFA / ferulic acid metabolites	Su et al., 2023
Banxia Xiexin Decoction (BXD)	Targets oxidative stress and steroidogenesis	↓ androgen synthesis; ↓ oxidative stress	FOS, JUN, MAPK3, TP53, HSP90AA1; CYP17A1	Shen et al., 2025
Dingkun Pill (DKP)	Regulates androgen biosynthesis	↓ Testosterone; ↓ LH/FSH ratio; ↑ ovulation	CYP17A1	Jin et al., 2022; Cai et al., 2022
Modified Guishen Pill	Regulates sex hormones and oxidative stress	↓ IR; ↓ oxidative stress; ↓ apoptosis	Hormone signaling / metabolomics network	Tian et al., 2022
Erchen Decoction (ECD)	Regulates steroid-biosynthesis genes	↑ Ovarian function; ↑ follicular development	CYP17A1, HSD11B1, AKR1D1, HSD11B2	Zhang et al., 2025
HeQi San (HQS)	Modulates gut flora; reduces inflammation	↓ IR; ↓ inflammation; ↓ androgens	Microbiota composition	Li et al., 2024

### 4.3 TCM monomers, mechanisms, and therapeutic effects in PCOS

In addition to multi-herb prescriptions, several TCM monomers/extracts demonstrate targeted benefits in PCOS. In rodent models and a pilot human study, artemisinin derivatives lowered testosterone, improved cycles and PCOS features by promoting LONP1-mediated degradation of CYP11A1, thereby suppressing ovarian androgen synthesis (Liu et al., 2024; Tysoe, 2024). Meta-analysis of animal/early human data shows quercetin reduces insulin, glucose, cholesterol and testosterone, improving histology and endocrine indices (Su et al., 2024). Mechanistic work indicates modulation of PI3K/AKT, steroidogenesis genes, and improved folliculogenesis (Shah et al., 2023). In PCOS RCTs and clinical studies, berberine improved insulin resistance, lipid profile, body composition and hormone status, in some endpoints comparable to or exceeding metformin (Wei et al., 2012; Mishra et al., 2022). A double-blind RCT showed resveratrol reduced ovarian/adrenal androgens with concurrent improvement in insulin sensitivity (Banaszewska et al., 2016). In RCTs, 12-week curcumin improved fasting glucose/insulin, HOMA-IR, lipids and modulated PPAR-γ/LDLR expression; a double-blind trial also reported favorable glucose/insulin and androgen endpoints (Jamilian et al., 2020; Heshmati et al., 2021). Collectively, these findings highlight that bioactive TCM monomers can simultaneously modulate metabolic, endocrine, and inflammatory networks, representing promising adjuncts or lead compounds for developing multi-targeted therapies against PCOS.

## 5 Future research directions and clinical application challenges in dietary intervention and gut microbiota regulation

Individual differences significantly affect the effectiveness of dietary interventions in the management of PCOS. Differences in gut microbiota composition among individuals can lead to varying responses to the same nutritional intervention. When developing dietary intervention protocols, it is essential to consider individual specificity to enhance their efficacy. Current traditional Chinese medicine research on PCOS primarily focuses on animal experiments. The conduct of long-term follow-up studies and large-scale clinical trials is significant, as this not only helps validate the long-term efficacy and safety of dietary interventions and microbiota regulation but also reveals differences in intervention responses among different PCOS subtypes. The development of novel microbiome-based formulations represents a cutting-edge area for enhancing gut microbiota in patients with PCOS. These formulations include probiotics and prebiotics, which can effectively regulate gut microbiome balance. However, current regulatory frameworks for probiotic formulations remain inadequate, potentially impacting their clinical safety and efficacy. Therefore, establishing standardized management protocols for novel microbiome-based formulations will be a critical measure for enhancing the effectiveness of PCOS management in the future.

## 6 Conclusion

Dysbiosis of the gut microbiota plays a crucial role in the pathogenesis of PCOS. Changes in dietary patterns can effectively regulate the endocrine and metabolic status of patients with PCOS. This finding offers a new perspective on the complexity of PCOS and provides novel approaches for future therapeutic strategies. Dietary patterns, combined with microecological interventions using probiotics and prebiotics, show promising potential in improving clinical symptoms and metabolic abnormalities in PCOS. These interventions not only restore the balance of the gut microbiota but also improve insulin sensitivity, reduce inflammation, and subsequently influence hormone levels and ovarian function.
